# The complete mitogenome dataset of the heptageniid mayfly *Afronurus levis* (Ephemeroptera: Heptageniidae: Ecdyonurinae) from South Korea

**DOI:** 10.1016/j.dib.2024.111137

**Published:** 2024-11-13

**Authors:** Seong Duk Do, Dae-Yeul Bae, Jae-Hun Kim, Jae-Sung Rhee

**Affiliations:** a Department of Marine Science, College of Natural Sciences, Incheon National University, Incheon, South Korea; b Institute of Korea Eco-Network, Daejeon, South Korea; c Research Institute of Basic Sciences, Incheon National University, Incheon 22012, South Korea; d Yellow Sea Research Institute, Incheon, South Korea

**Keywords:** Complete mitogenome, Heptageniid, *Afronurus levis*, Phylogenetic analysis

## Abstract

The Heptageniidae family stands out as one of the most abundant and widespread among mayflies, distinguished by its unique mitochondrial characteristics in intergenic spacers and duplication of transfer RNA (tRNA) genes. In this dataset, we present the complete mitochondrial genome sequence of *Afronurus levis* (Navás, 1912), a member of the Heptageniidae family of mayflies. The total length of the *A. levis* mitogenome was determined to be 15,362 base pairs, encompassing 13 protein-coding genes (PCGs), 23 tRNA genes, and two ribosomal RNA genes. An additional *trnM* gene was detected in the *A. levis* genome, consistent with observations in other Heptageniid species.

The base composition of the *A. levis* mitogenome was determined to be 31.7 % A, 32.7 % T, 14.1% G, and 21.5 % C. Phylogenetic analysis using PCGs confirmed that the newly sequenced *A. levis* mitogenome clusters within the genus *Afronurus*, showing closer proximity to that of *A. drepanophyllus*.

Specifications Table

**Table:** 

Subject	Genomics
Specific subject area	Insecta, Mitogenome, Bioinformatics, Phylogenetic analysis
Data format	Raw and Analyzed
Type of data	Table: Mitogenome data used in the phylogenetic analysis Figures: Image of *Afronurus levis*, feature of the circular map of the *A. levis* mitochondrial genome, image of putative secondary structures for the tRNA genes of the *A. levis* mitochondrial genome, image of codon frequency and relative synonymous codon usage (RSCU) in protein coding genes (PCGs) of the *A. levis* mitogenome, phylogenetic tree analysis Supplementary figures: Image of repeat sequence in the control region, image of the average depth of coverage
Data collection	Genomic DNA source: An individual specimen Genomic DNA isolation: DNeasy Blood and Tissue Kit (Qiagen, Hilden, Germany) Library construction: TruSeq DNA Sample Preparation Kit (Illumina, San Diego, CA, USA) Sequencing: Illumina HiSeq platform (150 bp; HiSeq X ten; Illumina, San Diego, CA, USA) Quality check: Fast QC v.0.11.9, Trimmomatic v.3.9 Assembly and annotation: NOVOplasty v.4.2.1, MITOS2 webserver Mitogenome map construction: Proksee webserver Phylogenetic tree analysis: DAMBE v.7.3.5, IQ-TREE v.2.1.4, MAFFT v.7.490, trimAl v.1.2 PCGs composition analysis: MEGA v.11.0 tRNAs structure prediction: tRNAscan-SE webserver Phylogenetic tree visualization: Figtree v.1.4
Data source location	Location: Anyang Stream (37°25′N, 126°55′E) City: Anyang (Gyeonggi Province) Country: South Korea
Data accessibility	Repository name: NCBI database of nucleotide Data identification number: OQ863614 Direct URL to data: https://www.ncbi.nlm.nih.gov/nuccore/OQ863614.1 Repository name: NCBI BioProject Data identification number: PRJNA990950 Direct URL to data: https://www.ncbi.nlm.nih.gov/bioproject/PRJNA990950/ Repository name: NCBI BioSample Data identification number: SAMN36278127 Direct URL to data: https://www.ncbi.nlm.nih.gov/biosample/SAMN36278127/ Repository name: Sequence Read Archive Data identification number: SRR25143774 Direct URL to data: https://www.ncbi.nlm.nih.gov/sra/SRR25143774

## 1 Value of the Data

• The mitogenome data presented in this study provides the initial complete mitochondrial genome analysis of Afronurus levis, marking the first report of this species within the genus Afronurus collected in South Korea.
• Although Heptageniidae is one of the most abundant and widespread families of mayflies, comprising over 600 described species, only a few complete mitochondrial genomes are currently available. Therefore, this information will prove valuable in enhancing our understanding of the geographical distribution and genetic diversity of mayflies, with a particular focus on the genus Afronurus.
• Mayflies exhibit a variety of mitochondrial gene organization patterns, including tRNA gene duplication and intergenic spacer variation. Therefore, this data will be instrumental in elucidating whether the mitochondrial structure is unique or conserved within each lineage.
• In addition to caddisflies and stoneflies, mayflies are among the three most commonly utilized indicators of the health status of aquatic environments due to their juvenile life (nymph) in the water and high sensitivity to pollution. Therefore, the mitochondrial DNA information presented here will be valuable for its application in environmental monitoring, such as through the use of techniques like environmental DNA analysis.

## 2 Background

Ephemeroptera is considered a primitive group of winged insects that possess ancestral morphological characteristics (e.g. extra appendages and wings that do not fold flat over the abdomen) and a distinctive prometamorphosis development pattern [[1], [2], [3]]. Currently, Ephemeroptera comprises 42 families and 478 genera, with approximately 3778 species [4]. Among these families, Heptageniidae stands out as one of the most species-rich families within the Ephemeroptera [5]. Heptageniidae includes three subfamilies: Ecdyonurinae, Heptageniinae, and Rhithrogeninae. Despite efforts to determine the phylogenetic relationships of Heptageniidae using both morphological characteristics and genomic information [[5], [6], [7], [8], [9], [10], [11]], certain aspects remain controversial. One such controversy lies in whether Heptageniidae is sister to Isonychiidae [7,8,10]. Additionally, phylogenetic relationship between the three subfamilies of Heptageniidae is also a subject of debate, particularly regarding which subfamily is sister to Heptageniinae [5,10,12]. Phylogenetic analyses and gene rearrangement patterns have suggested that Ecdyonurinae and Rhithrogeninae are sister groups to the exclusion of Heptageniinae [11]. Given their unique morphologies, diversity, ecological dynamics, and complex evolutionary histories, the acquisition of sufficient genomic data is crucial for further taxonomic relationships within this insect group.

Members of the mayfly genus *Afronurus* Lestage, 1924 play significant trophic roles in freshwater faunas and are widely distributed in the Afrotropical and Oriental regions. The genus was originally described for the South African species *Ecdyonurus peringueyi* Esben-Petersen, 1913. Another genus, *Cinygmina* Kimmins, 1937, was established for *Cinygmina assamensis* Kimmins, 1937, from India, but it was later synonymized as *Afronurus assamensis* [12,13]. Currently, *Afronurus* comprises 66 species [14]. Although *Afronurus* species have been consistently found in the Afrotropical and Oriental regions [15], no genomic information from this genus is available from South Korea. To address this knowledge gap, our study aimed to sequence and characterize the entire mitogenome of *A. levis*, providing a reference for inferring robust phylogenetic relationships and population genomics in heptageniids.

## 3 Data Description

**Fig. 1:**
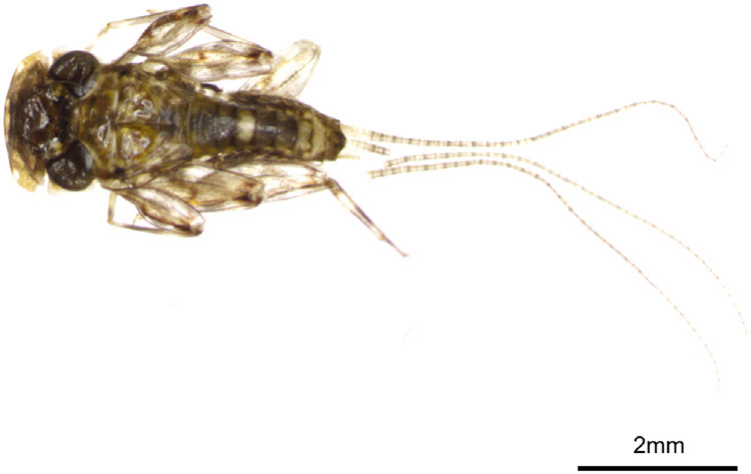
Species reference image of *Afronurus levis* collected from the Anyang Stream. The photo was taken by the authors.

The juvenile stage (nymph) of *A. levis* was characterized by four spots along the anterior edge of the head, with an additional pair of spots positioned between both compound eyes (Fig 1) [16].

**Fig. 2:**
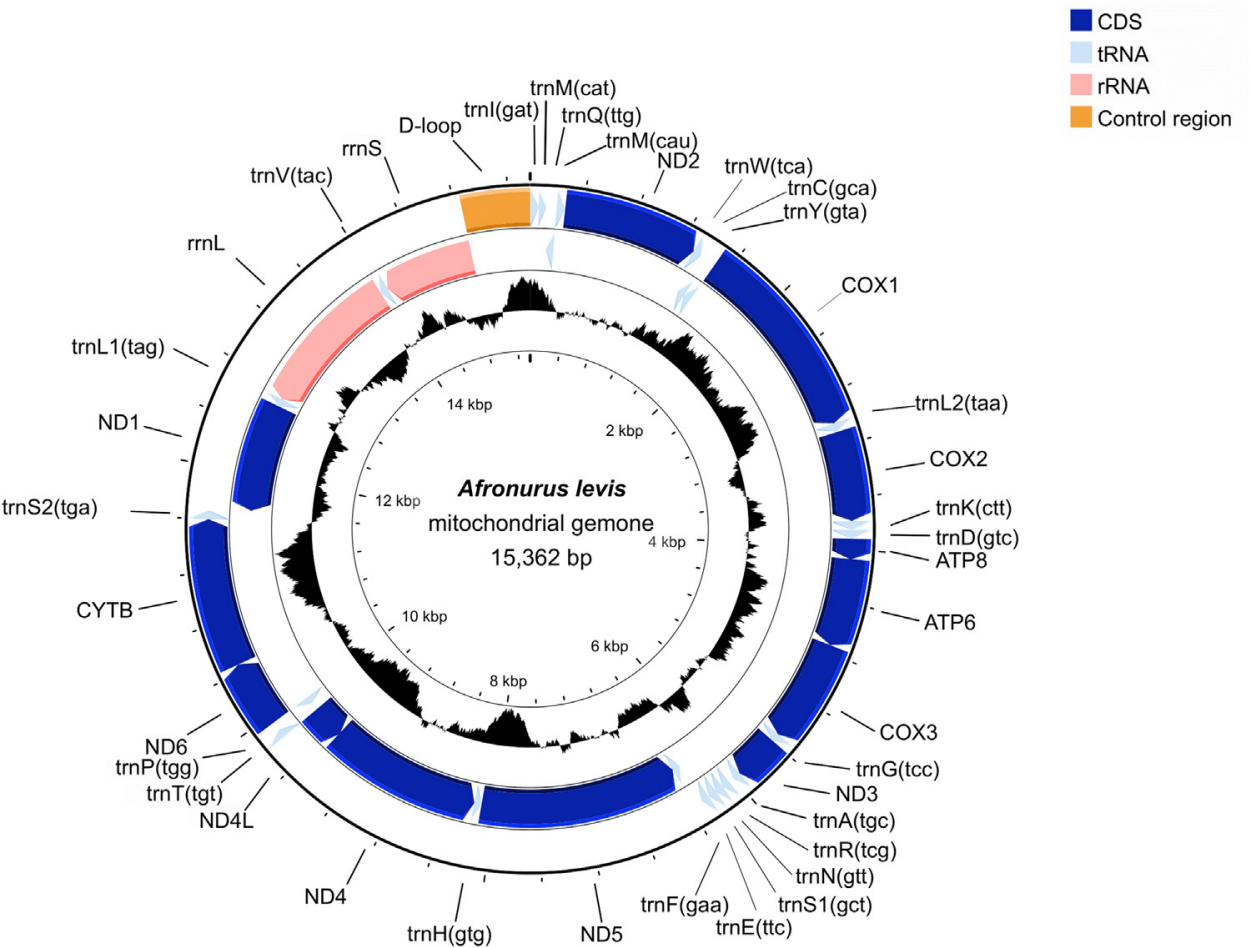
Circular map of the assembled *Afronurus levis* mitochondrial genome, consisting of 13 protein-coding, 23 transfer RNA, and two ribosomal RNA genes. Genes encoded on the reverse strand and forward strand are illustrated inside and outside the circles, respectively.

The complete mitogenome of *A. levis* is 15,362 bp long (GenBank accession no OQ863614) and contains 13 protein-coding genes (PCGs), two ribosomal RNA (rRNA) genes (12S and 16S), and 23 transfer RNA (tRNA) genes (Fig 2). The newly sequenced nucleotide sequence of the *cox1* gene in the mitogenome exhibited a 99.8 % similarity with a partial *cox1* sequence of *A. levis* previously deposited in GenBank (MN608816).

**Fig. 3:**
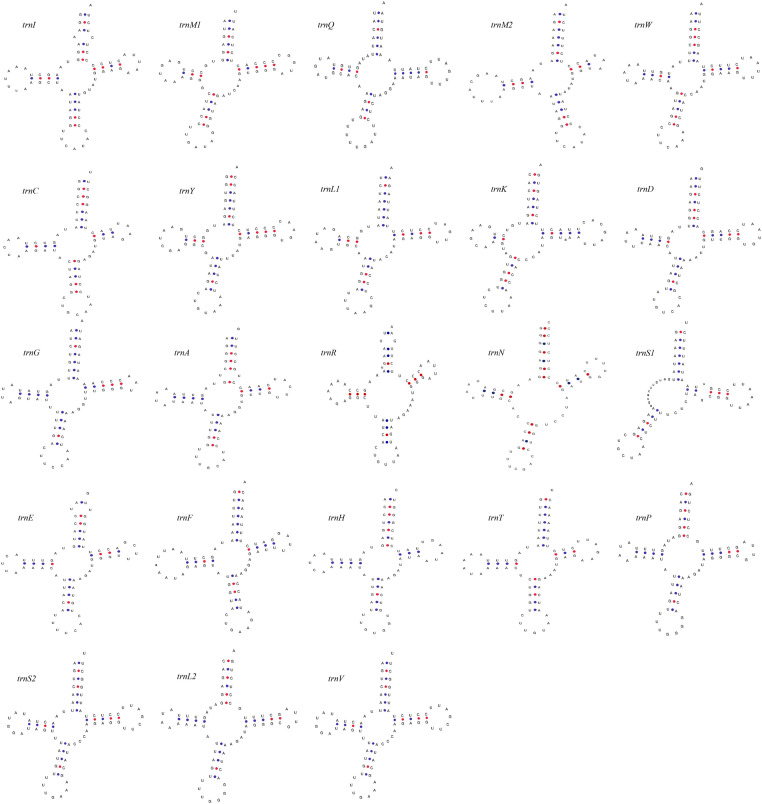
Putative secondary structure of 23 tRNAs for *Afronurus levis* mitogenome.

**Fig. 4:**
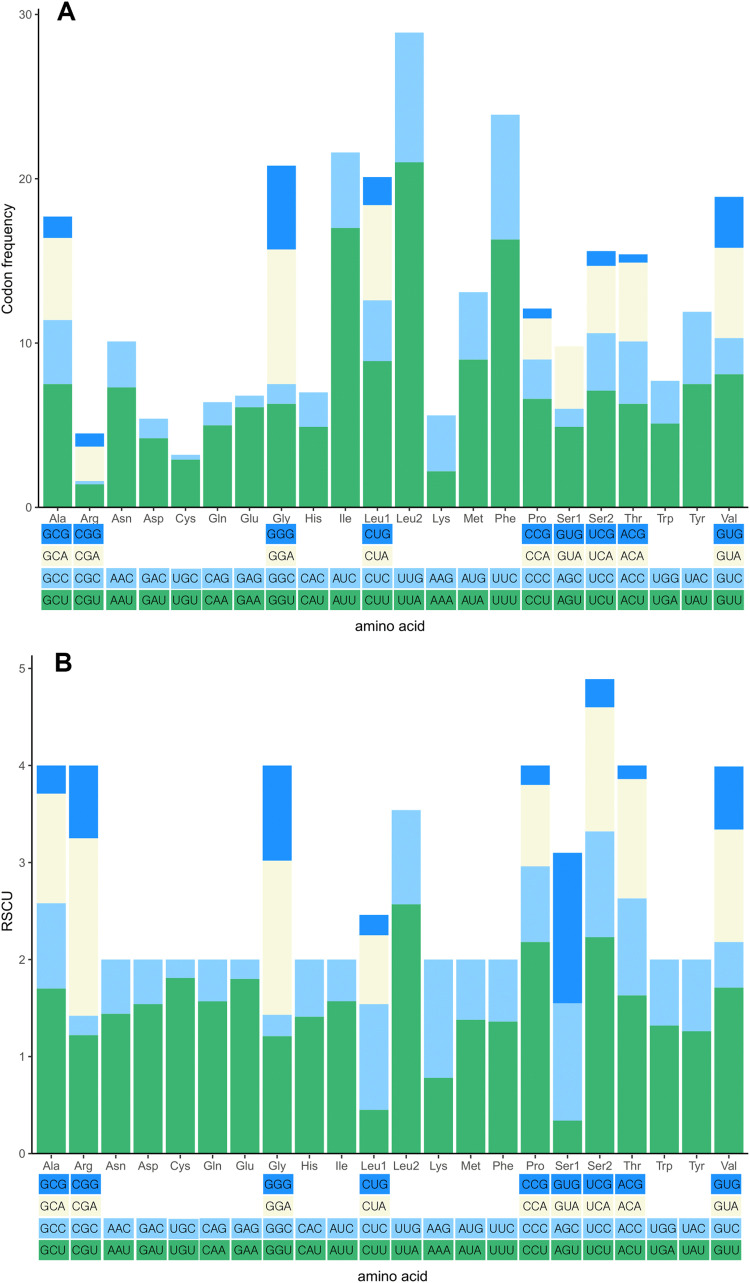
Codon frequency (A) and relative synonymous codon usage (RSCU) (B) in the 13 PCGs of *A. levis*.

**Fig. 5:**
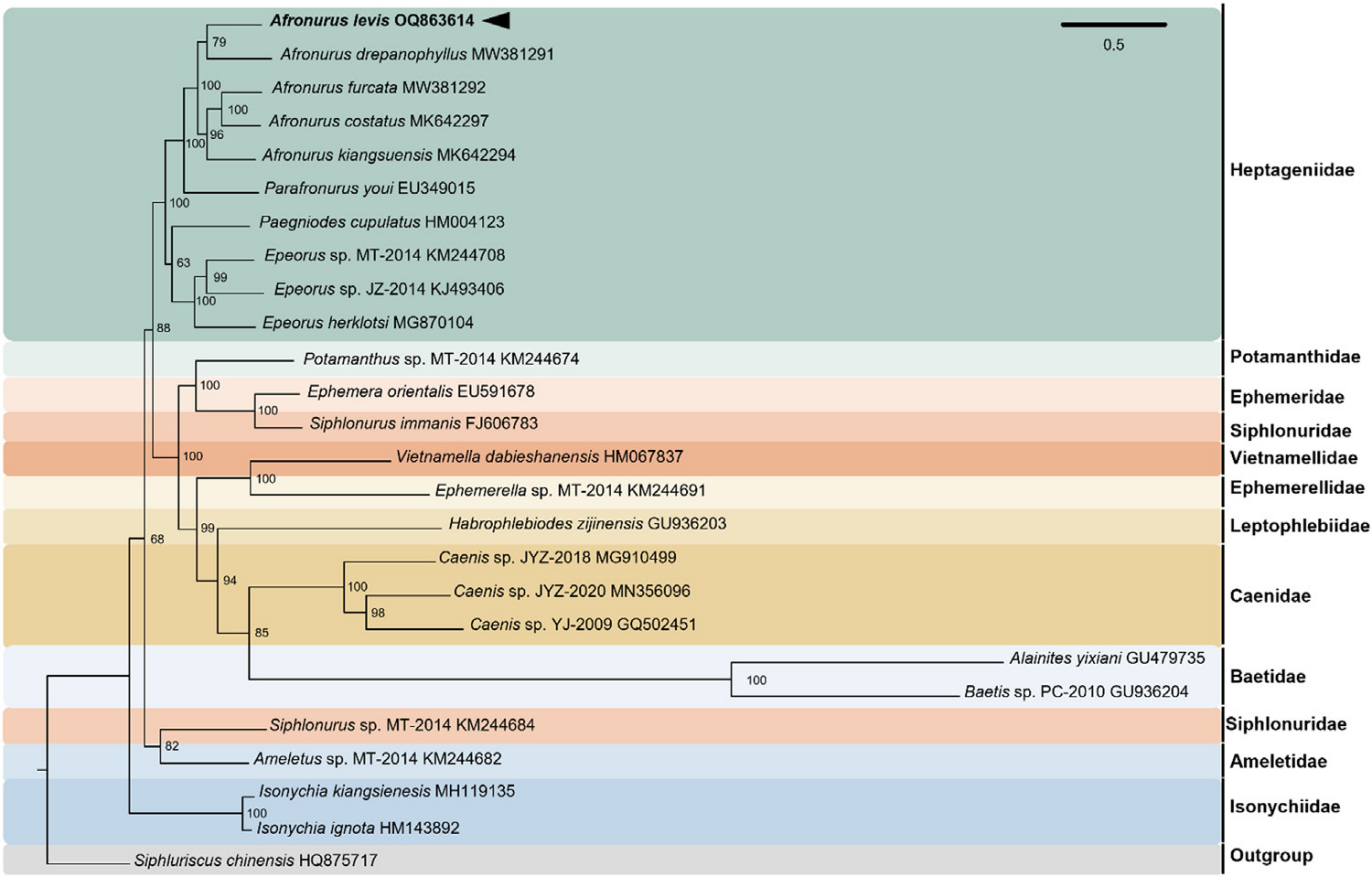
Maximum-Likelihood (ML) phylogenetic tree of 26 published complete mitogenomes of Heptageniidae, including that of *Afronurus levis*, based on the concatenated nucleotide sequences of protein-coding genes (PCGs). The numbers on the branches indicate ML bootstrap percentages. DDBJ/EMBL/Genbank accession numbers for published sequences are incorporated. The bold texts represent the mayfly analyzed in this study. Details on the mitogenome data are appended in Table 1.

Twenty-five genes were encoded on the majority strand, with the remaining genes located on the minority strand. An additional *trnM* gene (*trnI*-*trnM*-*trnQ*-*trnM*) followed by the *ND2* gene and a conserved intergenic spacer (IGS) were identified in *A. levis* mitogenome. The IGS is 39bp long and located between *trnA* and *trnR*. These patterns are typical among species of the subfamily Ecdyonurinae, as further supported by the phylogenetic tree in Fig. 5, where *A. levis* is placed within the same clade as Ecdyonurinae [11]. The putative secondary structures of the entire tRNAs identified in the *A. levis* mitogenome are depicted in Fig. 3. Consistent with previous studies on Heptageniidae species, all tRNAs exhibited a cloverleaf secondary structure, except for tRNA^Ser(AGN)^, which lacked a dihydrouridine (DHU) arm due to a deletion [17]. Nine PCGs began with the conventional ATN start codon, while *nad2, nad5*, and *atp8* used GTG, and *cox1* started with CCG. Nine PCGs terminated with conventional stop codons (TAA or TAG), whereas *cox2, nad4*, and *nad5* had incomplete stop codons (T-). These truncated stop codons are commonly observed across various organisms and are completed by post-transcriptional polyadenylation [18]. The 13 PCGs comprised a total of 11215 nucleotides, encoding 3738 amino acids. The five most frequently used codons were UUA (Leu2), AUU (Ile), UUU (Phe), AUA (Met), and CUU (Leu1) (Fig. 4a). The RSCU revealed a strong preference for T and A in the third codon position, a pattern common in *Afronurus* species (Fig. 4b). Overall, the GC content was 35.5 %, with the following percentages for individual nucleotides: 31.7 % A, 32.7 % T, 14.1 % G, and 21.5 % C. Tandem repeats and other repeat sequences were identified within the control region (Fig. S1).

Phylogenetic analysis of PCGs derived from 26 species classified 12 families confirmed that *A. levis* is classified within Heptageniidae, being sister to other *Afronurus* species (Fig 5). Among the 12 families, Baetidae exhibited long branch attraction. However, the saturation test for the 13 PCGs showed that the Iss value (0.3683) was lower than Iss.c (0.8426) and Iss.Ac (0.6199). Therefore, no substitution saturation was detected in the PCGs.

## 4 Experimental Design, Materials and Methods

An individual specimen of *A. levis* (Fig 1) was sampled from the Anyang Stream (37°25′N, 126°55′E), Anyang City, Gyeonggi Province, South Korea. Partial tissues of *A. levis* were sampled for DNA extraction, registered in the collection of the Research Institute of Basic Sciences at Incheon National University, South Korea, under the Tissue No Insecta-61. Total genomic DNA was isolated from the tissue using the DNeasy Blood and Tissue Kit (Qiagen, Hilden, Germany) following the manufacturerʼs instructions. Next-generation sequencing was performed to obtain the complete mitogenome of *A. levis* using the HiSeq platform (150bp; HiSeq X ten; Illumina, San Diego, CA, USA) with previously established protocols [19]. Sequencing libraries were constructed using the TruSeq DNA Sample Preparation Kit (Illumina, San Diego, CA, USA) according to the manufacturerʼs protocol at Macrogen, Inc. (Seoul, South Korea). Library preparation sheared purified DNA samples by random fragmentation before ligating 5′ and 3′ adapters. Paired-end raw reads were cleaned using FastQC version 0.11.9 [20]. After demultiplexing, only matched index pairs were retained for further processing. Raw read data was trimmed to remove adapter sequences, low-quality reads, reads with >10 % unknown bases, or ambiguous bases using Trimmomatic v.3.9 [21]. From a total of 37,863,972 raw reads, 30,527,990 filtered reads were retained. De novo assembly using NOVOplasty v.4.2.1 obtained a circular contig of the *A. levis* mitogenome [22]. The average depth of coverage is provided in Supplementary Fig. 2. The consensus sequence was annotated using MITOS2 of the Galaxy platform [23]. Codon frequency and RSCU in 13 PCGs of *A. levis* were analyzed using MEGA 11 [24]. A mitochondrial genome map was created using Proksee webserver [25]. Putative tRNA structure was predicted using tRNAscan-SE webserver [26].

To assess the phylogenetic relationships of *A. levis*, a maximum-likelihood phylogenetic tree was constructed using aligned 13 PCGs (e.g. COX1, COX2, COX3, ATP6, ATP8, NAD1, NAD2, NAD3, NAD4, NAD4L, NAD5, NAD6, COB) from 26 Heptageniidae mitogenomes. *Siphluriscus chinensis* was used as an outgroup [27]. The detailed information used in phylogenetic tree is provided in Table 1. Sequences of 13 PCGs were aligned using l-INS-I algorithm with MAFFT v7.490 [28,29]. In each PCG, redundant gaps were eliminated by trimal v.1.2 [30]. Maximum-likelihood-based phylogenetic tree was constructed using IQ-TREE v2.1.4 [31]. Ultrafast bootstrapping support values for each node were calculated from 1000 replicates [32]. Tandem repeat sequences and duplicate sequences in control region were found using Tandem Repeat Finder v4.07 [33]. The saturation test was performed with DAMBE v.7.3.5 [34].

**Table 1:** Details on the mitogenome data used in the phylogenetic analysis.

Family	Genus	Species	Length (bp)	Reference
Heptageniidae	*Afronurus*	*Afronurus levis*	15,362	This study
	*Afronurus*	*Afronurus drepanophyllus*	15,242	Li et al. 2021
	*Afronurus*	*Afronurus furcata*	15,334	Li et al. 2021
	*Afronurus*	*Afronurus costatus*	15,883	Xu et al. 2021
	*Afronurus*	*Afronurus kiangsuensis*	15,519	Xu et al. 2021
	*Parafronurus*	*Parafronurus youi*	15,481	Zhang et al. 2008
	*Paegniodes*	*Paegniodes cupulatus*	15,721	Li et al. 2021
	*Epeorus*	*Epeorus* sp.	15,456	Tang et al. 2014
	*Epeorus*	*Epeorus* sp.	15,338	Unpublished
	*Epeorus*	*Epeorus herklotsi*	15,502	Gao et al. 2018
Potamanthidae	*Potamanthus*	*Potamanthus* sp.	14,937 (P)	Tang et al. 2014
Ephemeridae	*Ephemera*	*Ephemera orientalis*	16,463	Song et al. 2019
Siphlonuridae	*Siphlonurus*	*Siphlonurus immanis*	15,529	Unpublished
Vietnamellidae	*Vietnamella*	*Vietnamella dabieshanensis*	15,761	Unpublished
Ephemerellidae	*Ephemerella*	*Ephemerella* sp.	15,256	Xu et al. 2020
Leptophlebiidae	*Habrophlebiodes*	*Habrophlebiodes zijinensis*	14,355 (P)	Unpublished
Caenidae	*Caenis*	*Caenis* sp.	15,254	Cai et al. 2018
	*Caenis*	*Caenis* sp.	15,392	Xu et al. 2020
	*Caenis*	*Caenis* sp.	15,351	Unpublished
Baetidae	*Alainites*	*Alainites yixiani*	14,589	Unpublished
	*Baetis*	*Baetis* sp.	14,883	Unpublished
Siphlonuridae	*Siphlonurus*	*Siphlonurus* sp.	14,745	Tang et al. 2014
Ameletidae	*Ameletus*	*Ameletus* sp.	15,141 (P)	Tang et al. 2014
Isonychiidae	*Isonychia*	*Isonychia kiangsienesis*	15,456	Ye et al. 2018
	*Isonychia*	*Isonychia ignota*	15,105	Unpublished
Siphlonuridae	Siphluriscus	*Siphluriscus chinensis*	16,616	Li et al. 2014

*Note:* The abbreviation P means a partial mitogenome.

## Limitations

Not applicable.

## Ethics Statement

The authors have read and follow the ethical requirements for publication in Data in Brief and confirming that the current work does not involve human subjects, animal experiments, or any data collected from social media platforms.

## Credit Author Statement

S.D. Do: conceptualization, methodology, software, writing; D.-Y. Bae: methodology, reviewing; J.-H. Kim: methodology, reviewing; J.-S. Rhee: conceptualization, supervision, reviewing, and editing.

## Data Availability

NCBI database of nucleotideAfronurus levis mitochondrion, complete genome (Original data).

## Appendix Supplementary materials

Image, application 1
