# Supplementary material for: The complete mitogenome dataset of the heptageniid mayfly Afronurus levis (Ephemeroptera: Heptageniidae: Ecdyonurinae) from South Korea
Source: Data Brief. 2024 Nov 13;57:111137. doi: 10.1016/j.dib.2024.111137 (PMC11647139; doi:10.1016/j.dib.2024.111137)

**< Supplementary Information >**

**The complete mitogenome dataset of the heptageniid mayfly *Afronurus levis* (Ephemeroptera: Heptageniidae: Ecdyonurinae) from South Korea**

Seong Duk Do^a^, Dae-Yeul Bae^b^, Jae-Hun Kim^b^, Jae-Sung Rhee^a,c,d,*^

^a^Department of Marine Science, College of Natural Sciences, Incheon National University, Incheon, South Korea; ^b^Institute of Korea Eco-Network, Daejeon, South Korea; ^c^Research Institute of Basic Sciences, Incheon National University, Incheon 22012, South Korea; ^d^Yellow Sea Research Institute, Incheon, South Korea

Corresponding author’s email: jsrhee@inu.ac.kr

**Supplementary figure 1. Tandem repeat and duplicate sequence in the control region of *Afronurus levis* mitogenome.** Nucleotides provided below represent sequence information for each type.


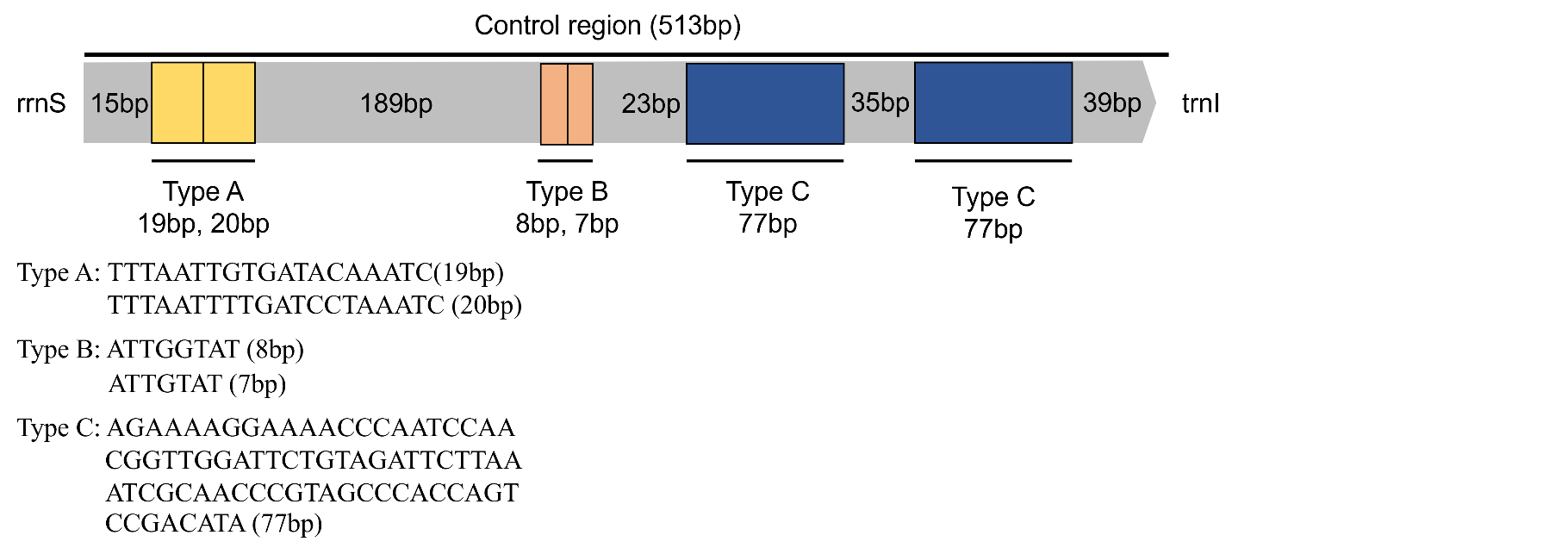


**Supplementary figure 2. The coverage depth of the mitochondrial genome of *Afronurus levis*.**


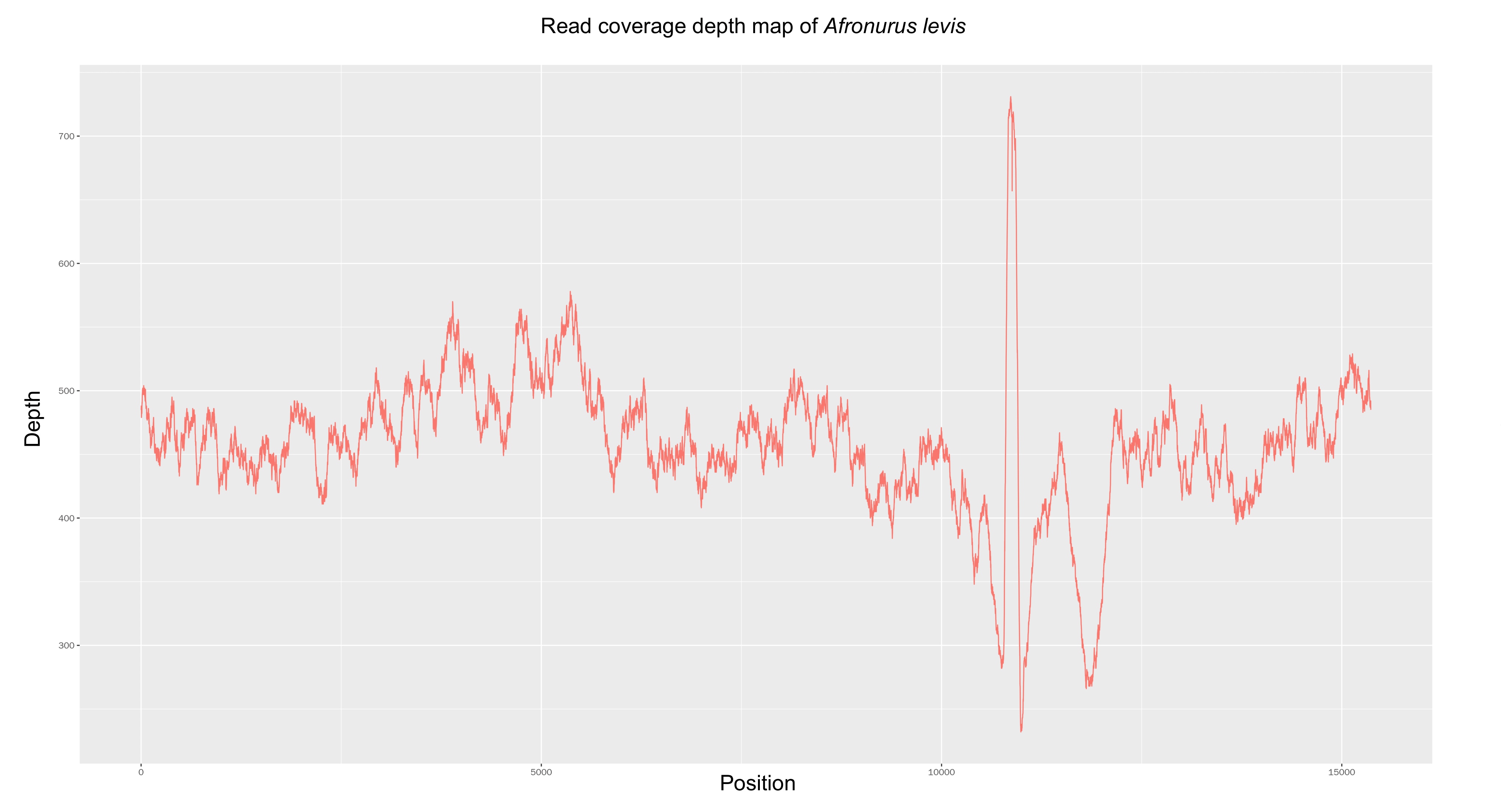

Supplement: Supplementary file 1 [file mmc1.docx]
